# Editorial: Pharmacological advancements of novel natural-based nanomedicines

**DOI:** 10.3389/fphar.2026.1823081

**Published:** 2026-03-19

**Authors:** Marios Spanakis, Ana Isabel Fraguas, Sofia Papadimitriou

**Affiliations:** 1 Department of Social Medicine, School of Medicine, University of Crete, Heraklion, Greece; 2 Department of Pharmaceutics and Food Technology, Faculty of Pharmacy, Complutense University, Madrid, Spain; 3 Prolepsis Institute of Preventive Medicine Environmental and Occupational Health, Athens, Greece

**Keywords:** drug research and development, nanomedicine, nanopharmaceutical materials, nanopharmacology, natural-based biomaterials, pharmacology

Natural-based nanomedicines are increasingly recognized as a transformative approach for enhancing drug delivery, therapeutic precision, and safety profiles through the integration of nanotechnology with biologically derived materials (Spanakis et al., 2014; Li et al., 2022; Azahar et al., 2025; Jahangirian et al., 2017; Manzari-Tavakoli et al., 2024; Ayub et al., 2025; Parvin et al., 2025; Salama et al., 2020). This Research Topic set out to highlight cutting-edge scientific advances that address key pharmacological, biopharmaceutical, and safety challenges in developing advanced drug delivery systems from natural-derived materials, encompassing bioavailability enhancement, sustainable design, and translational strategies.

The central aim was to bridge foundational nanoformulation science with translational pharmacology and regulatory considerations, while presenting multidisciplinary insights into Research Topic such as nano-encapsulation of phytochemicals, biosafety and toxicity assessment, computational prediction of pharmacokinetics, and pragmatic approaches for clinical translation (Parvin et al., 2025; Tripathi et al., 2024; Kim et al., 2025; Ahmad et al., 2022; Saidi et al., 2025). The collected contributions highlight advances spanning metabolic disorders, oncology, wound healing, neurological injury, and emerging nanovesicle technologies, while emphasizing sustainability, mechanistic insight, and translational feasibility.

A central objective of this Research Topic was to demonstrate how nano-engineering can overcome intrinsic limitations of natural compounds that otherwise constrain therapeutic application (Andreani et al., 2024; Han et al., 2022). The study of Moin et al., “*Piperine-loaded solid lipid nanoparticles: a promising nano-phytomedicine for the treatment of non-alcoholic fatty liver disease*” illustrates this principle by encapsulating piperine into solid lipid nanoparticles to enhance oral bioavailability and prolong hepatic circulation. The formulation exhibited sustained release behavior and significantly improved metabolic and hepatic parameters in a hyperlipidaemic mouse model compared with free piperine, underscoring the pharmacological value of nano-encapsulation in improving therapeutic exposure and efficacy.

The therapeutic potential of natural nanoformulations is further exemplified in hepatic disease management. In the work of Hu et al., “*Ursolic acid drug-drug nanocrystals ameliorate cholestatic liver injury via inhibiting oxidative stress and regulating bile acid metabolism*,” co-assembled nanocrystals composed of ursolic acid and α-tocopherol succinate demonstrated enhanced dissolution, improved bioavailability, and restoration of liver function in a cholestatic injury model. Mechanistically, the formulation modulated oxidative stress pathways and bile acid metabolism, highlighting the importance of rational nano-design in targeting disease-specific molecular networks. These findings collectively reinforce the role of nanocarriers in enabling synergistic pharmacodynamics and improving treatment outcomes in liver pathology (Padmanaban et al., 2025; Zhu et al., 2025).

Natural nanomedicine also holds promise in regenerative and neurological contexts. The study “*Unveiling the effects of Rosa canina oligosaccharide liposome on neuropathic pain and motor dysfunction following spinal cord injury in rats*” from Ahmadpour et al., investigates a liposomal nanoformulation designed to mitigate oxidative stress following spinal cord injury. Treatment improved sensory-motor function, enhanced antioxidant defenses, and promoted neuronal survival, demonstrating how nano-delivery systems can support neuroprotection and functional recovery. These findings contribute to the growing evidence supporting antioxidant-based nanotherapies in neurological disorders (Talaat et al., 2025; Li et al., 2020).

Chronic wound management represents another major translational challenge addressed within this Research Topic. The review work from Yadav et al., “*Therapies and delivery systems for diabetic wound care: current insights and future directions*” discusses current understanding of diabetic wound pathophysiology and examines how nano-enabled delivery systems enhance the therapeutic performance of plant-derived bioactive formulations. By integrating phytochemicals with polymeric or metallic nanocarriers, targeted delivery, antimicrobial action, and tissue regeneration may be improved. The review also contextualizes emerging nano-therapies within ongoing clinical and patent developments, bridging experimental innovation with clinical implementation pathways (Psarrou et al., 2023; Ansari and Darvishi, 2024; Kammona et al., 2024; Ahmad et al., 2025; Siafaka et al., 2025).

In oncology, natural nanomaterials are increasingly explored for targeted therapy and diagnostic applications. The review from Pandey et al., “*Biosynthesis of silver nanoparticles from plant extracts: a comprehensive review focused on anticancer therapy*” provides an extensive overview of plant-mediated silver nanoparticle synthesis and their emerging roles in cancer detection and treatment. By leveraging phytochemicals as reducing and stabilizing agents, green-synthesized nanoparticles offer a sustainable strategy to enhance selectivity and reduce toxicity compared with conventional treatments. Complementing this perspective, the research article of Cui et al., “*Plant-derived extracellular nanovesicles: a promising biomedical approach for effective targeting of triple negative breast cancer cells*” presents experimental data considering the anticancer activity of Citrus limon-derived extracellular nanovesicles. These vesicles demonstrated cellular uptake, suppression of proliferation and migration, and modulation of PI3K/AKT and MAPK/ERK signaling pathways in triple negative breast cancer models. Overall, both findings highlight the convergence of green chemistry, and nanotechnology, as a promising direction for future therapeutic developments in oncology (Bakhshan et al., 2025; Zhang et al., 2026; Zhou et al., 2026; Ion et al., 2021; M et al., 2025; Zango et al., 2023; Fraguas-Sá et al., 2025; Spanakis et al., 2024; Vizirianakis et al., 2016).

Beyond experimental advances, understanding research trajectories is essential for guiding future innovation. The bibliometric analysis “*Unveiling the dynamic trends of plant-derived exosome nanovesicles-based theranostics: through bibliometric and visualized analysis*” from Cao et al., maps the years of scientific activity in plant-derived nanovesicle research. By identifying dominant research themes, contributing institutions, and emerging hotspots, this work provides a strategic overview of the field’s evolution and highlights opportunities for interdisciplinary collaboration and translational advancement (Elayaperumal et al., 2025; Barathi et al., 2024; Agrahari and Agrahari, 2018; Xing et al., 2025; Barkat et al., 2020).

Across these contributions, several cross-cutting themes emerge. First, nano-encapsulation consistently enhances pharmacokinetic performance and therapeutic stability of natural compounds, reinforcing the importance of formulation science in maximizing biological activity (Goktas et al., 2020; Shaji and Jayasri, 2023; Sadr et al., 2025; Papadimitriou et al., 2008; Umar et al., 2025). Second, sustainability and biosafety remain essential considerations, particularly in the context of green synthesis and long-term biological interactions of nanomaterials (Bai et al., 2024; Xuan et al., 2023; Oladipupo et al., 2025; Fadeel et al., 2018). Third, mechanistic understanding linking nano-structure to biological function is increasingly emphasized, supporting rational design strategies for targeted therapy (Moyano and Rotello, 2011; Liu and Tang, 2017; Chehelgerdi et al., 2023; Shan et al., 2024; Han et al., 2025). Finally, translational progress will depend on integrating predictive modeling, standardized characterization, and regulatory frameworks to bridge preclinical success with clinical application (Ozbek et al., 2024; Rodríguez-Gó et al., 2025; Bobo et al., 2016; Desai et al., 2025).

In summary, this Research Topic highlights the extent and momentum of natural-based nanomedicine research, spanning fundamental formulation science to disease-specific therapeutic applications. The collected works demonstrate that natural nano-formulations can enhance drug performance, modulate disease pathways, and offer sustainable alternatives to conventional therapies. Continued interdisciplinary collaboration and rigorous translational strategies will be essential to fully realize the clinical potential of these emerging pharmacological technologies.
